# Influence of growth media components on the antibacterial effect of silver ions on *Bacillus subtilis* in a liquid growth medium

**DOI:** 10.1038/s41598-018-27540-9

**Published:** 2018-06-19

**Authors:** Ilse De Leersnyder, Leen De Gelder, Isabel Van Driessche, Pieter Vermeir

**Affiliations:** 1 Department of Green Chemistry and Technology, Laboratory of Chemical Analysis (LCA), Faculty of Bioscience Engineering, Ghent University, Ghent, Belgium; 2 Department of Biotechnology, Laboratory for Environmental Technology, Faculty of Bioscience Engineering, Ghent University, Ghent, Belgium; 3 Department of Inorganic and Physical Chemistry, Sol-gel Center for Research on Inorganic Powders and Thin film Synthesis (SCRiPTS), Faculty of Science, Ghent University, Ghent, Belgium

## Abstract

Numerous studies have investigated the antibacterial effect of both silver ions and silver nanomaterials on a large diversity of environmentally and clinically relevant bacteria. However, contradictory results are reported in which inhibition concentrations were varying by a 10-fold. This study investigated whether this variance in results could be attributed to the difference in experimental conditions, especially the microbial growth medium. *B. subtilis* was exposed to 500 µg L^−1^ Ag^+^ in liquid growth media with different concentrations of some commonly used media components: tryptone, yeast extract, Cl^−^, and S^2−^. The toxic effect was investigated by means of three complementary analysis techniques: (i) analyzing the growth curves obtained by optical density measurements, (ii) using flow cytometry, and (iii) by transmission electron microscopy. The silver ion toxicity towards *B. subtilis* decreased as more tryptone, yeast extract, or S^2−^ was present. This study demonstrates that the medium composition, rarely acknowledged as an important experimental factor in bacterial toxicity studies, has a profound impact on the observed silver toxicity towards *B. subtilis*.

## Introduction

Silver and its salts have been used for centuries for its effective antibacterial, antifungal, and antiviral properties in preserving water or food and preventing infections of wounds. More recently, silver nanoparticles (AgNPs) were developed, resulting in a remarkable increase in silver usage in various fields in industry^1–3^. For example, the increasing bacterial resistance against current antibiotics led to the rise of interest in some innovative AgNPs applications in the pharmaceutical and medical industry^4–6^. The antimicrobial effect of AgNPs, silver salts, and colloidal silver is mainly due to the continuous release of ionic silver (Ag^+^), which is the main bioactive species of silver^7–10^. However, the antibacterial mode of action of Ag^+^ is still unclear and different target sites were described. Studies reported that silver ions interfere with thiol groups present in proteins and enzymes inhibiting their functioning^11,12^. Possible alternative mechanisms, include interference with DNA molecules which whereby lose their replication abilities^11,13^, and inflicting structural cell changes and membrane damage that finally lead to cell death^11,14^. Despite the excellent properties of silver, the human and ecological risk of their release into the environment during the production and application has received increasing attention in many papers^15–17^. In order to predict the possible ecological and human toxicity of silver, as well as to verify the antimicrobial effect of new products and applications, a large number of scientific papers describe toxicity tests for AgNPs, Ag^+^, and colloidal silver towards a wide range of pure or mixed cultures of bacteria in both growth media or biological matrices. Nevertheless, results are often inconsistent, with varying reported inhibitory concentrations. Table 1 gives an overview of some selected Ag^+^ toxicity studies, performed on a single bacterial species (e.g. *E. coli*) in a classic liquid bacterial medium, in which inhibitory effects were observed with Ag^+^-concentrations ranging from 0.0635 mg L^−1^ to 100 mg L^−1^. Although these studies varied on several parameters in experimental conditions, we postulate that differences in the medium used in the toxicity test are at least partly responsible for the observed differences in inhibitory concentrations. As the toxicity of AgNPs, colloidal silver, and silver salts is mainly attributed to Ag^+^ ^18^, we investigated the influence of some commonly used growth media components on the antimicrobial effect of Ag^+^ on *B. subtilis* in this paper.

**Table 1:** Toxicity of *E. coli* against Ag^+^ in different liquid growth media.

Medium	Analysis technique	Concentration Ag^+^	Observed effect	Ref.
PBS^a^	FCM	0.2 mg L^−1^	Antibacterial efficacy of more than 90% after 2 h treatment	^14^
	Plate count	0.2 mg L^−1^	Antibacterial efficacy of 100% after 30 min treatment	^14^
	TEM	0.2 mg L^−1^	Cells are seriously damaged after 2 h treatment	^14^
Aqueous	Plate count	0.9 mg L^−1^ Ag^+^	Exponential decrease in cells with time (reduction of 10^7^–10^6^ CFU/mL in 24 h)	^27^
	TEM	0.9 mg L^−1^ Ag^+^	Various phases in the process of cell death were visible after 24 h treatment	^27^
MMD^b^	OD	2.5 mg L^−1^ Ag^+^	No growth after 24 h treatment	^28^
NB^c^	OD	0.635 mg L^−1^	Prolonged growth delay of ± 8 to 10 h, but after 24 h growth was observed	^31^
	Fluorescence assay	0.5 mg L^−1^	Growth inhibition of 100% after 24 h treatment	^26^
	MIC-screening	0.0635 ± 0.0318 mg L^−1^ Ag^+^ (MIC-value)	No growth after 24 h treatment	^44^
MH^d^	MIC-screening	6.35 mg L^−1^ (MIC-value)	No growth after 24 h treatment	^13^
LB^e^	TEM	6.35 mg L^−1^	Significant morphological changes after 4–12 h treatment	^11^
	OD	100 mg L^−1^ Ag^+^	No growth after 24 h treatment	^28^
	MIC-screening	3.5–5 mg L^−1^ Ag^+^ (MIC-value, depending on cell density)	No growth after overnight incubation	^30^
	OD	10 mg L^−1^ Ag^+^	No growth after 12 h treatment	^29^

^a^PBS = phosphate-buffered saline.

^b^MMD = modified minimal davis.

^c^NB = nutrient broth.

^d^MH = meuller hinton broth.

^e^LB = luria-bertani broth.

The composition of the different liquid growth media can be found in Table S1 of the Supplementary Information section.

## Results

### Development of a suitable testing medium

In order to test the influence of specific media components on the toxicity of Ag^+^ towards *B. subtilis*, a new growth medium (named IDL) was developed in which ions that form insoluble salts with Ag^+^, possibly leading to the unavailability of Ag^+^ and subsequent decrease of toxicity, were excluded. The composition of the IDL medium was similar to M9 medium but certain M9 salts which contain ions that form insoluble products with Ag^+^ were replaced by other salts. Solubility products constants (K_sp_) of Ag-bearing salts give a good indication of the potential forming insoluble salts or precipitates that can be formed. An overview of the K_sp_ values of the most relevant Ag-salts is shown in Table 2.

**Table 2:** Solubility products (K_sp_) of silver-containing solids at 25 °C^45,46^.

Compound (formula)	K_sp_
Silver sulfide (Ag_2_S)	6.30 × 10^−50^ mol^3^ L^−3^
Silver phosphate (Ag_3_PO_4_)	8.89 × 10^−17^ mol^4^ L^−4^
Silver chloride (AgCl)	1.77 × 10^−10^ mol^2^ L^−2^
Silver sulfate (Ag_2_SO_4_)	1.20 × 10^−5^ mol^3^ L^−3^
Silver nitrate* (AgNO_3_)	51.60 mol^2^ L^−2^

^*^Silver ions are added to the IDL medium under the form of the highly soluble silver nitrate.

The composition of the M9 medium and the developed IDL medium is shown in Table 3, from which it is apparent that because of the low K_sp_ value and thus poor solubility of AgCl, Cl^−^ bearing salts from the M9 medium were changed by SO_4_^2−^ or NO_3_^−^ salts. Based on the acid dissociation constants of H_2_PO_4_^−^ and HPO_4_^2−^ and the K_sp_ value of Ag_3_PO_4_, Ag_3_PO_4_ formation was not an issue in the performed set-up. In fact, disregarding the dissociation constants and assuming that all the H_2_PO_4_^−^ and HPO_4_^2−^ is transformed to PO_4_^3−^, Ag_3_PO_4_ formation could not be an issue at a concentration of 500 µg L^−1^ Ag^+^. Also, considering the Ag^+^ presence in the medium, the concentration of SO_4_^2−^ was theoretically too low for Ag_2_SO_4_ to be formed.

**Table 3:** Composition of M9 medium and IDL medium.

M9 medium^a^	IDL medium^b^
KH_2_PO_4_	KH_2_PO_4_
Na_2_HPO_4_.7H_2_O	Na_2_HPO_4_.12H_2_O
NaCl	Na_2_SO_4_
NH_4_Cl	(NH_4_)_2_SO_4_
MgSO_4_	MgSO_4_.7H_2_O
CaCl_2_	Ca(NO_3_)_2_.4H_2_O
Glucose	Glucose

^a^According to Sambrook and Russell^42^; modified salts are indicated by grayscale.

As *B. subtilis* was able to grow in the theoretically developed IDL medium, it proved to be usable for further experiments. By adding different media components (such as tryptone, yeast extract, Cl^−^ and S^2−^) to the IDL medium separately, the specific effect of each component on the toxicity of silver could be elucidated. A concentration of 500 µg L^−1^ Ag^+^ was used, because no growth of *B. subtilis* was observed in the IDL medium under the chosen experimental conditions at this concentration of silver (See Figure S1 of the Supplementary Information section).

### pH and EC of different growth media

The pH and EC of each medium were measured immediately after preparation. All pH values were in the range of 7.00 ± 0.10, with the exception of the media with 1 M Cl^−^ and 1.5 M Cl^−^ which had a slightly lower pH of 6.86 and 6.53 respectively. The EC of almost all the media was in the range of 10.00 ± 1.00 mS cm^−1^. Cl^−^ containing media showed ECs which were higher: 16.68 mS cm^−1^ for the 0.25 M Cl^−^, 27.90 mS cm^−1^ for the 1 M, and 71.60 mS cm^−1^ for the 1.5 M Cl^−^ containing medium. Obviously, the high amount of chlorine resulted in an increase in EC. The LB medium had a EC of 19.69 mS cm^−1^. All pH and EC values can be found in Table S2 of the Supplementary Information section.

### Evaluation of the antibacterial effect by OD measurements, FCM, and TEM

The influence of different media components on the toxicity of 500 µg L^−1^ Ag^+^ on *B. subtilis* was screened by OD measurements. For each medium, four treatments were set up: a bacterial culture with and without Ag^+^ and a sterile blank with and without Ag^+^. Each treatment was analyzed in quadruplicate and the average values of these four measurements ± standard deviations were calculated and plotted in function of time. To reduce clutter on the graph, the standard deviation was not shown at each time point but remained almost constant throughout the measurements. For all sterile blanks, a constant background value of ±0.100 was measured throughout the 48 h incubation.

**Figure 1:**
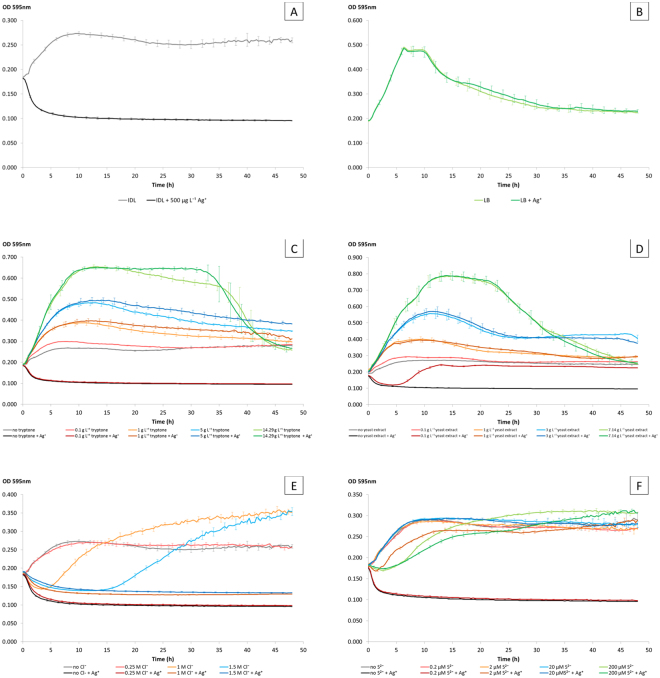
Growth of *B. subtilis* exposed to 500 µg L^−1^ Ag^+^ (compared with negative control) in IDL medium (**A**), LB medium (**B**), and IDL medium with different concentrations of tryptone (**C**), yeast extract (**D**), Cl^−^ (**E**), or S^2−^ (**F**). Error bars represent the standard deviation of quadruplicate analyses.

First, growth curves of *B. subtilis* with or without 500 µg L^−1^ Ag^+^ were obtained in the defined minimal IDL medium and the undefined complex LB medium (Fig. 1A,B). Growth of *B. subtilis* was inhibited in IDL medium (A) after Ag^+^ addition. In contrast, the growth curves obtained in LB (B) were similar with or without addition of Ag^+^. This experiment already clearly shows that there is a significant effect of the medium on silver toxicity. Subsequently, the influence of different media components on the toxicity of Ag^+^ (Fig. 1C–F) was determined separately, such that specific components could be attributed to this observation. The results show that the undefined complex components tryptone (C) and yeast extract (D) can totally diminish the antibacterial effect of 500 µg L^−1^ Ag^+^ at a concentration of 1 g L^−1^ tryptone or yeast extract. Moreover, addition of 0.1 g L^−1^ yeast extract already leads to a partial elimination of Ag^+^ toxicity: although the growth of bacteria was delayed with ca. 6 h compared to the control, the bacteria could still reach the stationary growth stage. Addition of Cl^−^ (E) did not lower the toxicity of Ag^+^ in the tested experiments. As a matter of fact, the controls lacking Ag^+^ with 1 M and 1.5 M Cl^−^ added showed an increased lag phase up to 5 and 15 h respectively. In contrast to Cl^−^, the addition of 20 µM S^2−^ (F) resulted in a complete elimination of Ag^+^ toxicity. In addition, a lower concentration of 2 µM S^2−^ also reduced the toxic effect but still, a growth delay was observed of about 2 h. At a higher concentration of 200 µM S^2−^, the untreated and Ag^+^ treated control showed a growth decline with a lag phase up to 4 h.

Subsequent to the OD measurements, FCM analyses were performed on samples from selected silver treatments, together with untreated control samples. The FCM outputs were acquired with triggering on FL2 (orange fluorescence). The sterile blank samples gave negligible signals. The FL1 (green fluorescence) versus FL3 (red fluorescence) cytograms were used to identify whether the bacterial cells were dead or alive. *B. subtilis* cells with intact membranes are considered to be alive, will have a green fluorescence, and appears therefore in the gate on the right side of the dot plot. In contrast, *B. subtilis* cells with damaged membranes are dead, will have a red fluorescence, and are consequently positioned in the left side of the cytogram.

**Figure 2:**
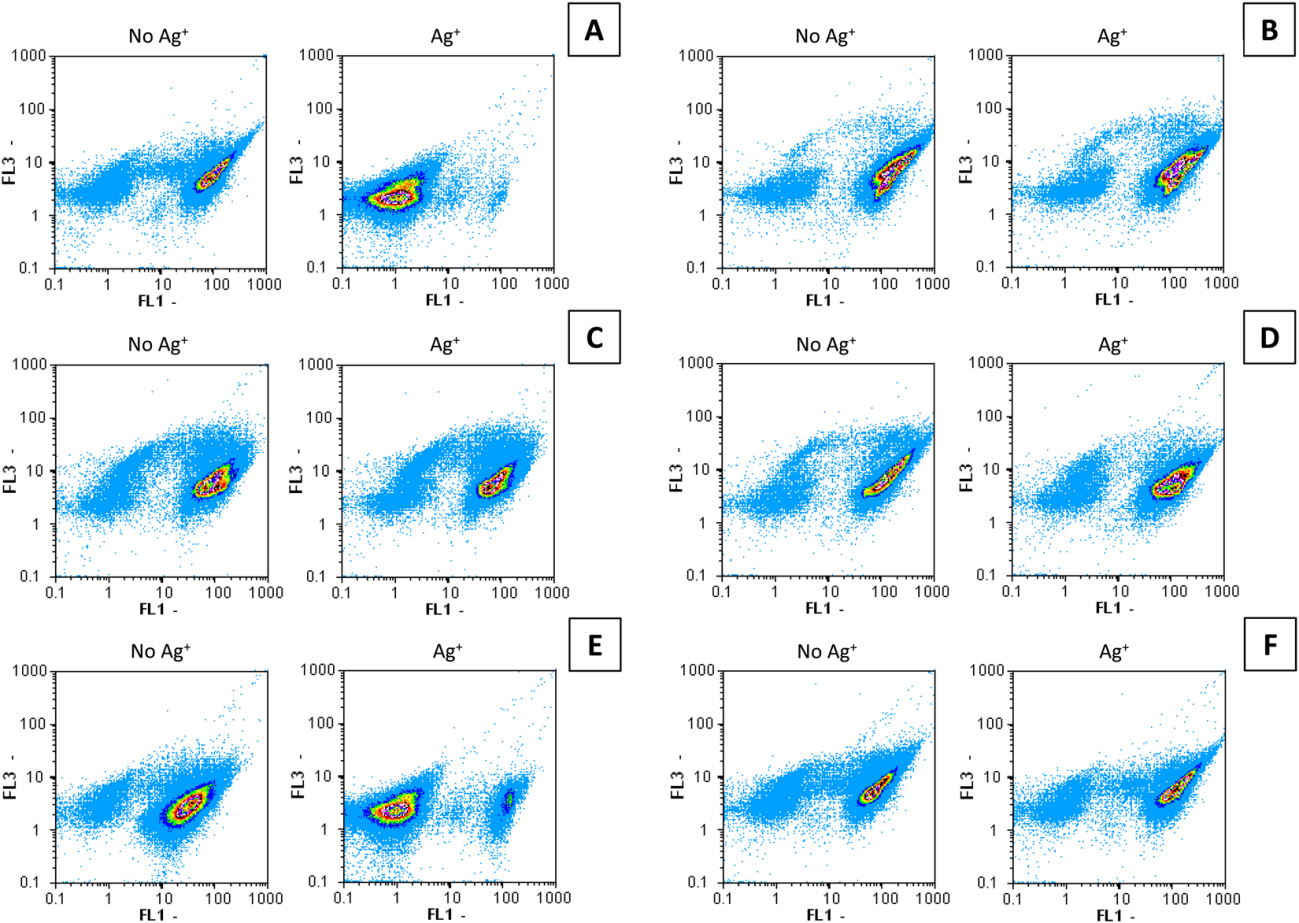
Membrane integrity of B. *subtilis* exposed to 500 µg L^−1^ Ag^+^ (compared with negative control) in IDL medium (**A**), LB medium (**B**), and IDL medium with 14.29 g L^−1^ tryptone (**C**), 7.14 g L^−1^ yeast extract (**D**), 0.25 M Cl^−^ (**E**), or 20 µM S^2−^ (**F**).

Figure 2 shows the FCM results of *B. subtilis* exposed to 500 µg L^−1^ Ag^+^ during 3 h in the different media (IDL, LB, and the adjusted IDL growth media). In all tested media without addition of Ag^+^, the proportion of intact cells was higher than 90% of the total number of bacteria. Bacterial cells treated with 500 µg L^−1^ Ag^+^ showed a significant lysis of more than 90% in the IDL medium (A) and IDL medium with 0.25 M Cl^−^ (E). Conversely, addition of 500 µg L^−1^ Ag^+^ didn’t lead to a significant lysis of the bacterial cells in the LB medium (B), and the IDL medium with 14.29 g L^−1^ tryptone (C), 7.14 g L^−1^ yeast extract (D), or 20 µM S^2−^ (F). In these media, more than 90% of the cells were considered to be alive despite of the presence of Ag^+^. These results are in agreement with the results obtained in OD measurements.

In the final stage, the OD measurements and FCM analyses were substantiated through TEM analyses of *B. subtilis* cells treated with Ag^+^ in the different media compared to the negative control, such that the external morphology and internal structure could be assessed. Similarly to FCM, TEM analysis was only performed on selected samples.

**Figure 3:**
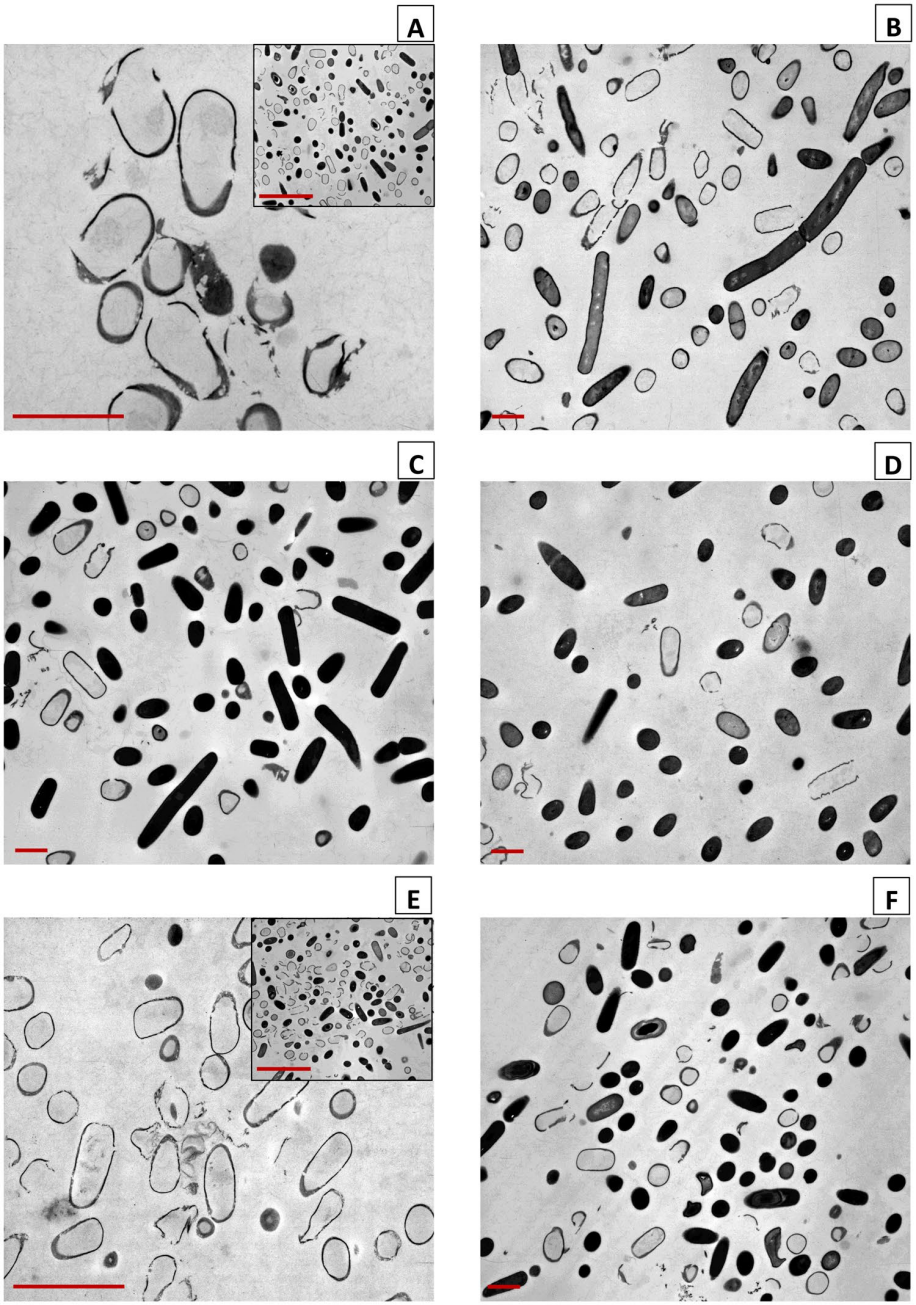
TEM images of *B. subtilis* cells treated with 500 µg L^−1^ Ag^+^ in IDL medium (**A**), LB medium (**B**), and IDL medium with 14.29 g L^−1^ tryptone (**C**), 7.14 g L^−1^ yeast extract (**D**), 0.25 M Cl^−^ (**E**), or 20 µM S^2−^ (**F**). The inserts in A and E show the negative control in IDL medium (**A**) and IDL medium with 0.25 M Cl^−^ (**E**). The scale bar in main images represents 1 µm, in the inserts the scale bar represents 4 µm.

*B. subtilis* is a gram positive, rod-shaped bacterium, arranged as single cells or in chains, and can form endospores. TEM photos of the 24 h cultures of 500 µg L^−1^ Ag^+^ treated *B. subtilis* in the six different media (Fig. 3) revealed that almost all cells were lysed and void of cytoplasmic fluid after Ag^+^ treatment in IDL medium (A) and IDL medium with 0.25 M Cl^−^ (E). However, in all other media (B, C, D, F) a greater fraction of the *B. subtilis* cells remained intact, which indicates that the toxic effect of Ag^+^ was diminished. In IDL medium with 20 µM S^2−^ some sporulating bacteria were observed. In the negative control (without Ag^+^) for both IDL (insert A) and IDL medium with 0.25 M Cl^−^ (insert E), TEM analyses showed the majority of the *B. subtilis* cells to remain intact. Therefore, it may be concluded that in these media, occurrence of cell lyses and empty cells is in fact due to the presence of Ag^+^. Also sporulation was observed in both media.

## Discussion

Up until now, silver toxicity studies rarely considered possible effects of the chemical composition of the medium in which the toxicity was tested. In order to increase our understanding in this respect, we investigated whether growth media components can affect the toxic effect of Ag^+^ perceived by bacteria, here specifically the gram-positive model organism *B. subtilis*. To avoid the presence of salts that can form insoluble products with Ag^+^, the IDL medium was developed. In order to evaluate the effect of growth medium components on the observed antimicrobial effect of Ag^+^, the composition of this defined minimal IDL medium was adjusted stepwise by adding different concentrations of some frequently present growth medium components: tryptone, yeast extract, Cl^−^, or S^2−^. Finally, the effect of Ag^+^ was also tested in LB, a commonly used undefined complex medium which contains both tryptone, yeast extract, and NaCl.

Tryptone is derived from the enzymatic digestion of casein out of milk and is widely used as protein source for bacterial growth. Yeast extract is, like tryptone, an undefined complex medium component providing peptides, carbon, sulfur, trace elements, nitrogen compounds, vitamin B, and a lot of other important growth factors^19,20^. In addition to these two undefined components, Cl^−^ and S^2−^ were considered. Bacteria need S^2−^ for vitamin and amino acid synthesis. Cl^−^ is often present as anion of salts which serve as nitrogen source or micro nutrient, or as NaCl, to maintain osmotic pressure^21,22^. As apparent from Table 2, Cl^−^ and S^2−^ are likely to form precipitates with Ag^+^ due to the low K_sp_’s. Moreover, *Bell et al*. showed that Ag^+^ are prone to precipitate with S^2−^ present in both inorganic and organic species^23^.

The EC, which is closely related to the osmotic pressure, and the pH are chemical properties of growth media that can affect bacterial growth^24,25^. To control the possible variances in growth due to a difference in pH and EC of the tested media, these two parameters were measured and the results were taken into account before drawing conclusions. The Cl^−^ containing media showed some deviating results and had a lower pH and higher EC compared to all other media. The observed growth delay in 1 M and 1.5 M Cl^−^ containing IDL media was therefore probably due to the high osmotic pressure. All other IDL and IDL adjusted media showed similar pH and EC values, such that the perceived toxicity of Ag^+^ for the bacterial cells could only be influenced by the ionic composition and/or the amount of organic matter.

In all experiments, *B. subtilis* was treated with a Ag^+^ concentration of 500 µg L^−1^. This concentration was chosen because results indicate that this amount of Ag^+^ gave a total growth inhibition for 48 h in IDL medium under the chosen experimental conditions. The growth curves of *B. subtilis* when exposed to different concentrations of Ag^+^ in IDL medium can be found in the Supplementary Information section (Figure S1).

Three different techniques (OD measurements, FCM & TEM) all corroborated the almost complete reduction of silver toxicity in IDL media by the addition of 14.29 g L^−1^ tryptone, 7.14 g L^−1^ yeast extract, or 20 µM S^2−^. Moreover, OD measurements revealed that silver toxicity was already diminished at 1 g L^−1^ tryptone and 1 g L^−1^ yeast extract. A concentration of 200 µM S^2−^ led, in both treatments with and without Ag^+^, to a growth delay. This S^2−^ concentration possibly exceeds the toxic level of S^2−^ for *B. subtilis*. Rather unexpectedly, surely when considering the K_sp_ of AgCl (Table 2), the addition of Cl^−^ did not reduce the antimicrobial effect of Ag. This could be explained by the fact that colloidal AgCl can also have an antibacterial effect^26^. Moreover, increasing Cl^−^ concentrations would lead to the increase in osmotic pressure and consequently growth delay, as previously described.

Previously obtained varying results in silver toxicity studies, as listed in Table 1, can be re-evaluated in the light of our results. Even though various experimental conditions are different between the studies, a general trend concerning growth media can be observed. Studies that were performed in a defined minimal medium, like PBS^14^, an aqueous medium^27^, and MMD^28^ observe a toxic effect at concentrations ranging from 0.2–2.5 mg L^−1^ Ag^+^. On the other hand, when undefined complex media like LB^11,28–30^ and MH^13^ were used, higher concentrations of Ag^+^ were necessary to obtain toxicity: 3.5–100 mg L^−1^ Ag^+^. At a lower concentration of 0.635 mg L^−1^ Ag^+^, only a growth decline with a lag phase up to 10 h was observed in the undefined complex NB medium^31^. Based on the results in this paper, the presence of tryptone, yeast extract, and S^2−^ are (at least partly) responsible for the observed difference between defined minimal versus undefined complex media. As shown in Table S1 of the Supplementary Information section, LB contains both tryptone and yeast extract. MH and NB contain similar compounds (extracts and casein hydrolysates) possibly leading to a similar effect and thus diminishing the toxic effect of Ag^+^.

Some recently published reviews^32–35^ already hinted at the importance of the possible interactions of medium components with silver when performing silver toxicity experiments (whether or not in ionic form) but an in depth study was missing up until now. Also in more complex systems, this phenomenon had been put forward in literature, for example in a study of *Çeçen et al*. which showed that the inhibitory effect of Ag^+^ on active sludge systems depended the type of organic matter in the feed, and peptone was suggested to form complexes with the Ag^+^ ions^36^. We believe that our stepwise approach by going from a defined minimal to an undefined complex medium was highly needed and can form a basis for further experiments. Moreover, our results can be extrapolated to more complex systems like biological matrices. In a broader perspective, when studying the now commonly used AgNPs, media components also have an remarkable influence on the stability of the particles, and hence their desired antibacterial properties^37–41^. Therefore, microbiologists studying (nano)silver toxicity should take possible medium effects into account when designing the experiments and interpreting the results.

## Conclusion

To the best of our knowledge, this is the first report to demonstrate the influence of media components on the antibacterial effect of silver ions towards *B. subtilis*. From this study, it has become clear that the effect of the test medium on silver toxicity towards *B. subtilis* cannot be neglected. In a broader perspective, researchers have to consider the possible chemical interference of media components with silver ions before drawing conclusions regarding silver toxicity towards possibly all bacteria. Previously obtained varying results in silver toxicity studies can be re-evaluated in the light of our results, thereby possibly resolving a point of conflict in the research field.

Based on our results, the IDL medium can be put forward as a standard medium for determining Ag^+^ toxicity studies toward *B. subtilis*. As a matter of fact, we would recommend researchers who are performing toxicity studies to Ag^+^ or other metal ions in general to interpret the Ksp values of poorly soluble salts that can be formed when choosing a medium or at least take it into account when interpreting the results. Not only when performing antibacterial studies but toxicity studies in general, for example to aquatic organisms like *Daphnia manga*, it is necessary to look at the interaction of the media with the tested component before taking conclusions about the effect of the component on the organism.

## Methods

All data generated or analysed during this study are included in this published article (and its Supplementary Information section).

### Preparation of Ag^+^ solution

0.1 M silver nitrate (AgNO_3_) (VWR) was diluted in autoclaved Milli Q® (Millipore, Merck) to a concentration of 10 mg L^−1^ Ag^+^. AgNO_3_ dilutions were always freshly prepared and kept in the dark.

### Preparation of different growth media

The composition of the used defined minimal medium, appointed as the IDL medium, was based on M9 medium^42^. A concentrated IDL salt solution was prepared by dissolving 15 g L^−1^ KH_2_PO_4_ (Sigma-Aldrich), 85.49 g L^−1^ Na_2_HPO_4_.12H_2_O (Merck), 3.04 g L^−1^ Na_2_SO_4_ (UCB), and 6.18 g L^−1^ (NH_4_)_2_SO_4_ (Merck) in autoclaved Milli Q®. This concentrated IDL salt solution was 5 times diluted and MgSO_4_.7H_2_O (Sigma-Aldrich) and Ca(NO_3_)_2_.4H_2_O (Acros Organics) were added as micronutrients to a final concentration of 2 mM and 0.1 mM respectively. Glucose (Merck) was added as the carbon source in 0.4% w/v to the final volume.

Some frequently present growth media components were added in different concentrations to the IDL medium. The tested concentrations of components added is shown in Table 4.

**Table 4:** Tested concentrations of components added to the IDL medium.

Component added^a^ to IDL medium	Tested concentration
Tryptone	0.1, 1, 5, or 14.29 g L^−1^
Yeast extract	0.1, 1, 3, or 7.14 g L^−1^
Cl^−^	0.25, 1, or 1.5 M
S^2−^	0.2, 2, 20, or 200 µM

^a^Tryptone (VWR), yeast extract (VWR), Cl^−^ as NaCl (VWR), S^2−^ as Na_2_S.9H_2_O (UCB).

The undefined complex Luria-Bertani (LB) medium was prepared by dissolving 10 g tryptone, 5 g yeast extract, and 10 g NaCl in 1 L of Milli Q®. The addition of 15 g agar (LabM) results in Luria-Bertani Agar (LBA), used for plates.

### pH and EC measurement of different growth media

The pH and electrical conductivity (EC) of each medium was measured by the inoLab® pH Level 1 pH-meter (WTW) with a SenTix® 81 electrode (WTW) and the K611 EC-meter (Consort) with a SK20T electrode (Consort) respectively.

### Preparation of *B*. subtilis cultures

The experiments were performed with *B. subtilis* LMG7135 (LMG culture collection, Ghent University, Ghent, Belgium). To obtain isolated colonies, the bacteria was streaked on LBA and incubated overnight at 37 °C for growth. A single colony was inoculated in 15 mL IDL medium in a sterile 50 mL Falcon® tube (Corning) which was incubated at 30 °C and 100 rpm during 20 h.

### Experimental treatments

Preparation of the different treatment mixtures was performed in sterile 15 mL Falcon® tubes (Corning). For treatments containing silver, 7 mL medium was mixed with 0.5 mL 10 mg L^−1^ Ag^+^ solution. For control treatments without silver, 0.5 mL autoclaved Milli Q® was added. After homogenizing, these mixtures were kept at room temperature overnight to reach chemical equilibrium. After equilibration, 2.5 mL of a 20 h old bacteria culture was added to the 15 mL tube and mixed vigorously. For sterile blanks, 2.5 mL IDL medium was added. Considering the dilution rate of 10 mg L^−1^ Ag^+^ solution (0.5 mL in a final volume of 10 mL), the treated bacteria were exposed to a 500 µg L^−1^ Ag^+^ concentration. The evaluation of the effect of Ag^+^ in the different growth media was performed by optical density (OD) measurements, flow cytometry (FCM), and transmission electron microscopy (TEM).

### Evaluation of the antibacterial effect by OD measurements

The growth was monitored by measuring each culture’s OD_595nm_ periodically by use of the Multiskan Ascent Platereader with Ascent software version 2.6 (Thermo Labsystems, Thermo Fisher Scientific). Immediately after combining the bacterial liquid culture (or blank IDL medium) with the equilibrated mix of medium and treatment, 200 µL of each mixture was dispensed in quadruplicate in a 96-well flat bottom multititer plate (Greiner Bio-one). After covering with a sterile Breath-Easy® film (Sigma Aldrich), the plate was incubated at 30 °C during 48 h. Growth in each well was assessed by measuring OD_595nm_ every 20 min. Every 10 min the plate was shaken at speed 60 rpm during 10 seconds. Growth curves are presented as the mean OD values ± standard deviations in function of time. To reduce clutter on the graph, the standard deviation was not shown at each time point.

### Evaluation of the antibacterial effect by FCM

After adding the bacterial liquid culture (or blank IDL medium) to the 15 mL Falcon - as previously described, the samples were incubated during 3 h at room temperature and regularly shaken, after which the samples were immediately diluted to a final volume of 1 mL by adding freshly prepared and filtered 0.85% NaCl (in Milli Q®) to obtain a suitable cell concentration. To distinguish the living cells from the dead ones, two different fluorescent dyes were used in this research. Because membrane integrity is considered to be one criterion distinguishing between dead and viable bacterial cells, 10 µL 0.5 mM Syto 13 (Life Technologies, Thermo Scientific) and 10 µL 1 mg L^−1^ Propidium Iodide (PI) (Life Technologies, Thermo Scientific) were added to the samples. The 0.5 mM Syto 13 was prepared by a ten-fold dilution of a 5 mM Syto 13 in DMSO solution with Milli Q®. The 1 mg L^−1^ PI was obtained by dilution with Milli Q® of a 10 mg L^−1^ PI stock solution in DMSO. Cells having intact membranes are considered to be alive while those with damaged membranes are dead. Syto 13 is a membrane permeable dye which stain live and dead bacteria while PI is a membrane impermeable DNA stain and therefore only interacts with dead cells^43^. After 15 minutes of incubation at 30 °C in dark conditions, the samples were ready for FCM analysis. The analyses were performed by the Chemunex® flowcytometer (CyTec GmbH) with Partex Flomax software (CyTex GmbH).

### Evaluation of the antibacterial effect by TEM

*B. subtilis* was exposed to 500 µg L^−1^ Ag^+^ in different growth media for 24 h on a shaker incubator at 30 °C and 100 rpm. As previously described, these treatments were performed in 15 mL tubes. After the treatments, the ultrastructural characteristics of *B. subtilis* were observed using TEM. Each bacterial suspension was first centrifuged for 15 min at 15000 $\times$ g and the obtained bacterial pellet was used for TEM sample preparation. Bacterial cells were subsequently fixed with 4 times diluted, 2 times diluted, and pure Karnovsky fixative consisting of 4% paraformaldehyde (EMS) and 2.5% glutaraldehyde (EMS) in 0.1 M sodium cacodylate buffer (EMS). Dilutions of the Karnovsky fixative were performed in 0.1 M sodium cacodylate buffer. The fixation step in the diluted fixative concentrations includes 20 min incubation at 4 °C and samples were continuously rotated. In pure Karnosky, 1 h rotation at 4 °C was required. After each fixation step, samples were centrifuged 5 min at 15000 × g and supernatant was removed. Cells were then rinsed 3 times 10 min in 0.134 M sodium cacodylate buffer, rotating during 20 min at 4 °C, and centrifuged 5 min at 15000 $\times$ g after each rinse step. Embedding took place in 1% low melding agarose, Ultra-pure TM (Invitrogen) at room temperature. Post-fixation occurs in ferrocyanide-reduced OsO_4_ during 1 h at room temperature. The ferrocyanide-reduced OsO_4_ consists of 1 mL 4% OsO_4_ (EMS), 3 mL 0.134 M cacodylate buffer, and 66 mg K_3_Fe(CN)_6_ (EMS). Afterwards, the cells were rinsed 4 times with distilled water and were stepwise dehydrated using increasing concentrations of ethanol (EMS) 15%, 30%, 50%, 70%, 90%, and 100%, 10 min each. Subsequently, the cells were infiltrated with the low-viscosity embedding Spurr (EMS) medium and polymerization was performed at 70 °C for 8 h. Samples were sectioned on a UC7 ultramicrotome (Leica) using a 35° diamond knife (Diatome) at room temperature. First semi-thin (0.5 µm) until the region of interest was reached and then ultra-thin (70 nm) sections were made. The ultra-thin cross-section was finally transferred to a copper TEM grid (Agar scientific) which was carbon coated afterwards with the EM ACE 600 C-coater (Leica). TEM analyses were performed at 60 kV with the TEM JEM1010 (Jeol). Pictures were digitalized using a Ditabis system.

## Electronic supplementary material

Supplementary information
