# Supplementary material for: Influence of growth media components on the antibacterial effect of silver ions on Bacillus subtilis in a liquid growth medium
Source: Sci Rep. 2018 Jun 19;8:9325. doi: 10.1038/s41598-018-27540-9 (PMC6008294; doi:10.1038/s41598-018-27540-9)
Supplement: Supplementary file 1 — Supplementary information [file 41598_2018_27540_MOESM1_ESM.docx]

**Influence of growth media components on the antibacterial effect of silver ions on *Bacillus subtilis* in a liquid growth medium**

Ilse De Leersnyder, Leen De Gelder, Isabel Van Driessche, and Pieter Vermeir

**Supplementary information**

**Supplementary tables**

| **Media** | **Composition (per 1 L H_2_O)** |
| --- | --- |
| PBS | 8 g L^-1^ NaCl  0.2 g L^-1^ KCl  1.44 g L^-1^ Na_2_HPO_4_  0.24 g L^-1^ KH_2_PO_4_ |
|  |  |
| MMD | 0.7 g L^-1^ K_2_HPO_4_  0.2 g L^-1^ KH_2_PO_4_  0.66 g L^-1^ (NH_4_)_2_SO_4_  0.5 g L^-1^ sodium citrate  0.1 g L^-1^ MgSO_4_.7H_2_O  3.31 g L^-1^ D-glucose |
|  |  |
| NB | 3 g L^-1^ beef extract  5 g L^-1^ peptone |
|  |  |
| MH | 2 g L^-1^ beef extract  1.5 g L^-1^ starch  17.5 g L^-1^ casein hydrolysate |
|  |  |
| LB | 10 g L^-1^ tryptone  10 g L^-1^ NaCl  5 g L^-1^ yeast extract |

**Table S 1:** Composition of liquid growth media TABLE 1.

| **Media** | **pH** | **EC (mS cm^-1^)** |
| --- | --- | --- |
| IDL | 7.06 | 9.53 |
|  |  |  |
| LB | 7.02 | 19.69 |
|  |  |  |
| IDL with 0.1 g L^-1^ tryptone | 7.05 | 9.69 |
| IDL with 1 g L^-1^ tryptone | 7.06 | 9.65 |
| IDL with 5 g L^-1^ tryptone | 7.06 | 10.08 |
| IDL with 14.29 g L^-1^ tryptone | 7.02 | 10.92 |
|  |  |  |
| IDL with 0.1 g L^-1^ yeast extract | 7.05 | 9.83 |
| IDL with 1 g L^-1^ yeast extract | 7.05 | 9.53 |
| IDL with 3 g L^-1^ yeast extract | 7.04 | 9.91 |
| IDL with 7.14 g L^-1^ yeast extract | 7.03 | 10.34 |
|  |  |  |
| IDL with 0.25 M Cl^-^ | 6.95 | 16.68 |
| IDL with 1 M Cl^-^ | 6.86 | 27.90 |
| IDL with 1.5 M Cl^-^ | 6.53 | 71.60 |
|  |  |  |
| IDL with 200 µM S^2-^ | 7.02 | 9.74 |
| IDL with 20 µM S^2-^ | 7.02 | 9.65 |
| IDL with 2 µM S^2-^ | 7.02 | 9.63 |
| IDL with 0.2 µM S^2-^ | 7.04 | 9.73 |

**Table S 2:** pH and EC of all tested media.

**Supplementary figures**


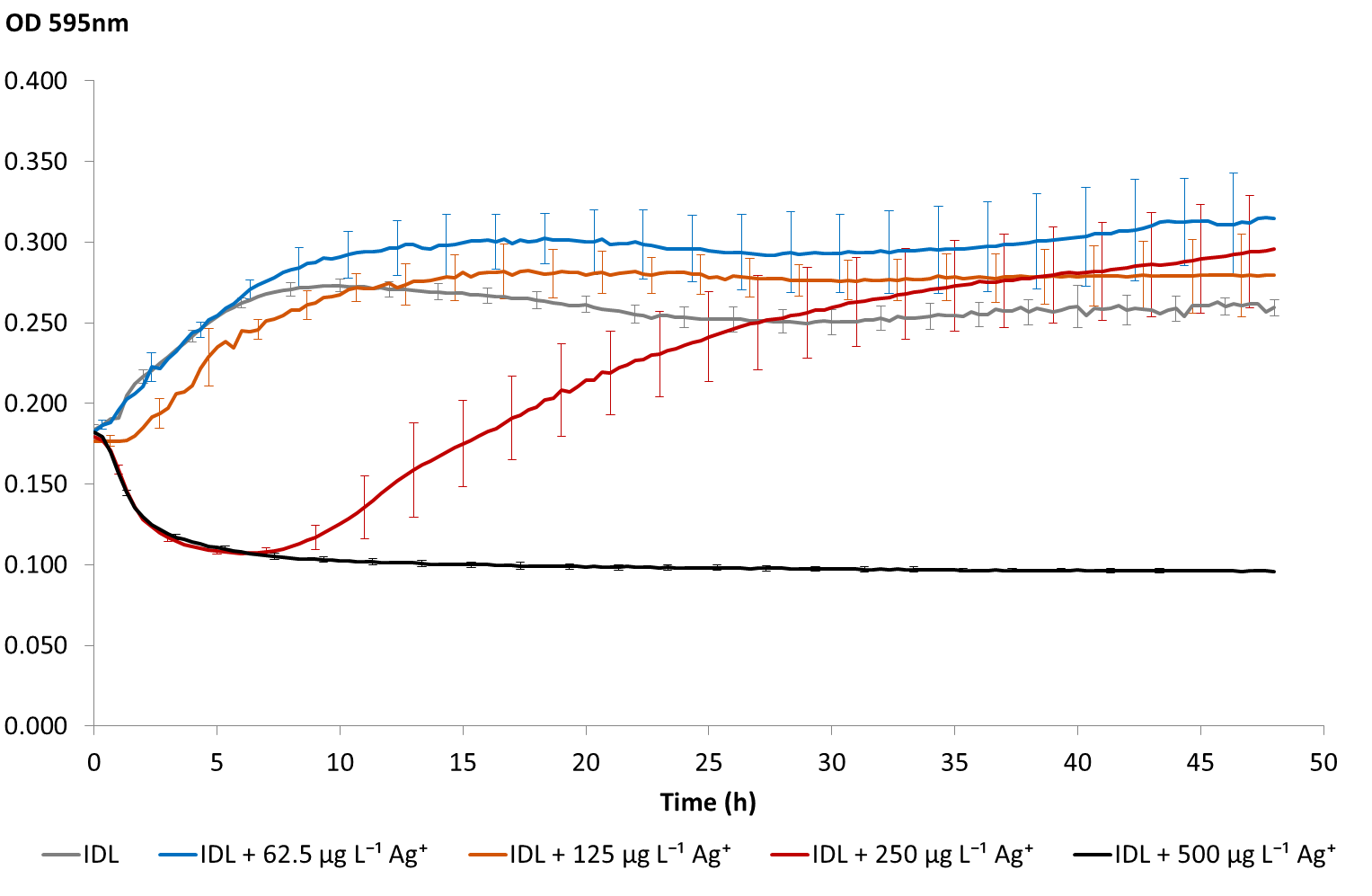


Figure S 1: Growth curves of *B. subtilis* exposed to different concentrations of Ag^+^ in IDL medium. No growth is observed during 48 h when exposed to 500 µg L^-1^ Ag^+^. Error bars represent the standard deviation of quadruplicate analyses.
